# Identical Strains of *Borrelia hermsii* in Mammal and Bird

**DOI:** 10.3201/eid1512.090792

**Published:** 2009-12

**Authors:** Robert J. Fischer, Tammi L. Johnson, Sandra J. Raffel, Tom G. Schwan

**Affiliations:** National Institutes of Health, Hamilton, Montana, USA (R.J. Fischer, T.L. Johnson, S.J. Raffel, T.G. Schwan); The University of Montana, Missoula, Montana, USA (T.L. Johnson)

**Keywords:** zoonoses, vertebrate host, Ornithodoros hermsi, bacteria, spirochetes, letter

**To the Editor:** On August 5, 1994, a northern spotted owl, *Strix occidentalis caurina*, was found dead in Kittitas County, Washington, USA (*1*). A thorough investigation and necropsy identified the probable cause of death to be a spirochete infection. The organisms were seen in sections of the bird’s liver with use of modified Steiner silver stain and microscopy. DNA was extracted from the infected liver, and PCR–DNA sequencing of the 16S ribosomal RNA (rRNA) locus identified the bacterium as a relapsing fever spirochete related most closely to *Borrelia hermsii* (*1*). The lack of additional data surrounding this case precluded Thomas et al. from concluding that this spirochete infecting the owl was *B. hermsii* (*1*). Yet, in a subsequent analysis using the intergenic spacer region, the owl spirochete was included with isolates of *B. hermsii* (*2*).

To investigate the distribution and prevalence of *B. hermsii* , during the summer of 2008, we began a study at Flathead Lake, Lake County, Montana, USA, where 9 persons have contracted relapsing fever since 2002 (*3*–*5*). A blood smear from 1 pine squirrel (*Tamiasciurus hudsonicus*) captured July 9 at Yellow Bay on the east shore of the lake (elevation 887 m; geographic coordinates 47°52′35′′N, 114°01′54′′W) contained spirochetes detected when stained with Giemsa and examined by microscopy (600× brightfield with oil immersion). Whole blood from the squirrel contained live spirochetes visible by dark-field microscopy, and ≈50 µL of this blood was injected intraperitoneally into a laboratory mouse. The next day, a few spirochetes were observed in the peripheral blood of the mouse, and during the next 3 days, the density of spirochetes increased. We used intracardiac puncture to collect blood from the mouse for spirochete isolation in BSK-H medium (Sigma-Aldrich, St Louis, MO, USA) and for analysis by PCR and DNA sequencing of multiple bacterial loci as described elsewhere (*4*,*6*).

The spirochetes observed in the squirrel’s blood failed to grow in BSK-H medium after passage in the laboratory mouse; however, we acquired DNA sequences from infected squirrel and mouse blood from PCR amplicons for 6 spirochete loci including *16S rDNA*, *flaB*, *gyrB*, *glpQ*, IGS, and *vtp*. Sequences for the loci were each aligned with homologous sequences from other borrelia in our collection, and each locus grouped the spirochete within the 2 genomic groups of *B. hermsii* described previously (*4*,*6*). The unique squirrel spirochete differed from all other *B. hermsii* identified in our previous studies; deep branches in each phylogram grouped the spirochete more closely with *B. hermsii* genomic group I than with genomic group II (data not shown).

Next, we compared the sequences from the squirrel spirochete with those available in the National Center for Biotechnology Information database (*www.ncbi.nlm.nih.gov*)*,* including those sequences reported for the spirochete found in the spotted owl (AY515269.1, AF116903.1, AF116904.1) (*1*,*2*). The 3 trimmed and aligned sequences for the *16S rDNA* (1,290 bases), *flaB* (467 bases), and IGS (665 bases) from the squirrel spirochete were identical to those of the owl spirochete; no base differences were found among the 2,422 bases compared. We also examined DNA extracted from the spotted owl’s liver during the first investigation (*1*) (provided by Alan G. Barbour). We successfully PCR amplified most of the *16S rDNA* and the complete *flaB*, *gyrB*, *glpQ,* and *vtp* genes from the owl spirochete DNA and determined their sequences. The complete sequences of the first 4 loci from the owl and squirrel spirochetes were identical and differed from all other *B. hermsii* sequences. A phylogram of the concatenated sequences totaling 5,188 bases demonstrated that the owl and pine squirrel spirochetes were identical and were divergent members of *B. hermsii* genomic group I (Figure).

**Figure:**
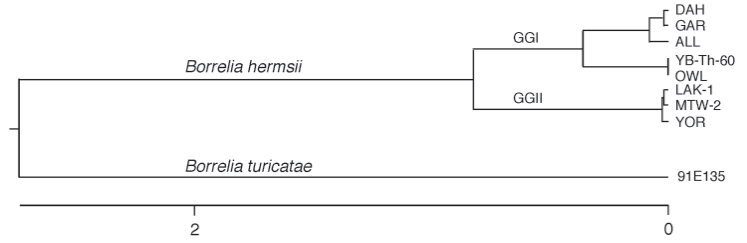
Phylogram based on the alignment of the concatenated DNA sequences containing the *16S rDNA*, *flaB*, *gyrB*, and *glpQ* loci for 6 isolates (DAH, GAR, ALL, LAK-1, MTW-2, and YOR) and infected tissues from the owl (OWL) and pine squirrel (YB-Th-60) of *Borrelia hermsii*. The same loci from *B. turicatae* 91E135 were used for the outgroup. New DNA sequences determined for the owl and pine squirrel spirochetes are available in GenBank (accession nos. GQ175059–GQ175068). Scale bar indicates number of base substitutions (×100).

Finding the same strain of *B. hermsii*, separated by ≈525 km, in a pine squirrel and a spotted owl demonstrates a broader geographic distribution and host range for this spirochete than what could have been envisaged previously. The possible role of birds as hosts for the vector *Ornithodoros hermsi* ticks has been demonstrated elsewhere (*4*). Given the ecologic overlap of pine squirrels and coniferous forest-dwelling birds, we believe that the previous finding of the infected spotted owl is likely not an isolated event. Instead, it may represent a tick–spirochete cycle for *B. hermsii* that includes a broader host range for this group of relapsing fever spirochetes than previously appreciated.
